# Assessment of Vascular Change Using Swept-Source Optical Coherence Tomography Angiography: A New Theory Explains Central Visual Loss in Behcet's Disease

**DOI:** 10.1155/2017/2180723

**Published:** 2017-05-09

**Authors:** Thanapong Somkijrungroj, Sritatath Vongkulsiri, Wijak Kongwattananon, Peranut Chotcomwongse, Sasivarin Luangpitakchumpol, Korrawan Jaisuekul

**Affiliations:** ^1^Department of Ophthalmology, Faculty of Medicine, Chulalongkorn University, Bangkok, Thailand; ^2^King Chulalongkorn Memorial Hospital, Bangkok, Thailand; ^3^Department of Ophthalmology, Phramongkutklao Hospital, Phramongkutklao College of Medicine, Bangkok, Thailand

## Abstract

**Objective:**

To evaluate retinal vascular structural change in ocular Behcet's using optical coherence tomography angiography (OCTA) and fluorescein angiography (FA).

**Methods:**

An analytic cross-sectional study of 37 eyes of 21 Behcet's uveitic patients was performed. Foveal retinal thickness (FRT), perifoveal hypoperfusion areas in superficial capillary plexus (SCP), and deep capillary plexus (DCP) were measured with swept-source optical coherence tomography and OCTA. FA images were used for assessing the vascular features and correlation.

**Results:**

Twenty-one patients were enrolled (52.4% males). The average age at onset was 36.7 ± 12.93 years. The median of disease duration was 5 years (1–25). FRT was 118.1 ± 52.35 *μ*m, which correlated with visual acuity (95% CI −60.47, −13.92). Using OCTA, the area of hypoperfusion in SCP (0.47 ± 0.17 mm^2^) was smaller than that in DCP (1.94 ± 3.87 mm^2^) (*p* < 0.001). Superficial to deep capillary plexus nonperfusion (SCP : DCP) ratio was 0.57 ± 0.27 which had the positive coefficient correlation with visual acuity (95% CI −0.644, −0.015).

**Conclusions:**

OCTA is an alternative noninvasive method to monitor macular ischemia in Behcet. Behcet's uveitis affects DCP more than SCP. Decreasing SCP : DCP ratio and decrease FRT correlates with poor visual acuity. Macular ischemia and DCP loss can be found early and can explain vision loss in Behcet.

## 1. Introduction

Behcet's disease, firstly described in 1937, is a chronic nongranulomatous occlusive vasculitis disease characterized by recurrent oral and genital ulcerations as well as ocular lesions. It can affect multiple organs including the heart, joints, and central nervous system. About 50% of the patients with Behcet's disease have ocular involvement [1].

Fluorescein angiography (FA) has been considered the gold standard to demonstrate the inflammatory activity in Behcet's uveitis. However, it is unable to demonstrate superficial and deep capillary vasculature separately. Furthermore, FA is a relatively invasive investigation and has a limitation in some patients due to dye usage.

Optical coherence tomography angiography (OCTA) is a noninvasive imaging modality to simulate angiographic images. Principle of OCTA is to detect decorrelation, the fluctuations of red blood cells signal from B-scan images, in each specific location of the retina. The advantage of OCTA is that it can demonstrate retinal vascular structure layer by layer, which allows physician to differentiate superficial capillary plexus (SCP), deep capillary plexus (DCP), and choriocapillaris (CC) separately [2].

There were many previous studies showing efficacies of OCTA in the evaluation of retinal vascular diseases such as age-related macular degeneration, diabetic retinopathy, retinal arterial and venous occlusion, sickle cell disease, and glaucoma [3–6]. The recent report using OCTA in ocular Behcet by Khairallah et al. showed a greater area of hypoperfusion in DCP compared to that in SCP [7]. In this study, we describe OCTA findings and propose the new theory explaining pathogenesis of central vision loss in patients with Behcet's disease.

## 2. Material and Methods

We performed an analytical cross-sectional study, approved by King Chulalongkorn Memorial Hospital (KCMH) research committee (IRB Approval number 45/59). All patients were given a full explanation of the study, and written informed consent was obtained prior to enrollment. The study protocol adhered to tenets of the Declaration of Helsinki.

Patients who were diagnosed with Behcet's uveitis, based on the international study group for Behcet's disease 1990 [8], presented at KCMH between May 2016 and June 2016 were enrolled. Patients who were under 18 years old or who had poor fixation for fundus evaluation using OCT were excluded. After informed consent was done, baseline parameters including age, sex, laterality, age of onset, disease duration, disease activity, and medical history were recorded. Ocular examination consisted of Snellen visual acuity converted to logarithm of minimum angle of resolution equivalent units (logMAR), intraocular pressure (IOP) by air-puff tonometry, OCT, OCTA, and FA was done.

DRI swept source OCT Triton (Topcon Corporation, Tokyo, Japan) was used to demonstrate perifoveal hypoperfusion area. 3×3 mm OCTA images centered at the macular region were performed by single experienced examiner. Swept-source optical coherence tomography angiography (SS-OCTA) was performed by using 100,000 Hz of A-scan, approximately one micron wavelength light source and deep signal penetration through the retina and choroid [9]. Time to acquire one image is approximately 4–6 seconds. By using Topcon's innovative OCTA processing method, the vascular structure is visualized. The pixel size generated by DRI OCT Triton (Topcon Corporation, Tokyo, Japan) is 320×320. The corresponding pixels in each position but different time were calculated. Then the en-face OCT angiography and structural en-face image will show in the program “IMAGEnet.”

For foveal retinal thickness (FRT), OCT B-scan image was manually selected and measured by using automate tool provided in IMAGEnet. The measurement was done between the inner border of internal limiting membrane and inner border for retinal pigment epithelium.

**Figure 1:**
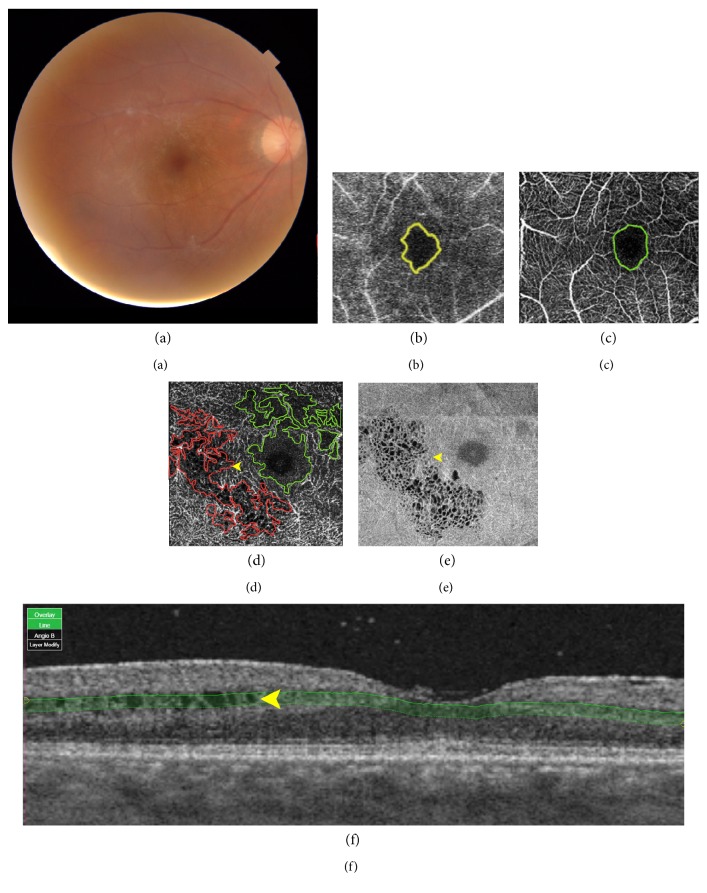
Multimodality imaging in the Behcet uveitis eye with 20/20 visual acuity complain about central visual loss. (a) Fundus photography shows generalized retinal artery attenuation. (b) Fluorescein angiography (FA) demonstrates hypoperfusion area measured in the early phase of FA (yellow area). (c) Superficial capillary plexus (SCP) slap in the optical coherence tomography angiography (OCTA) demonstrates hypoperfusion area in SCP (green area). (d) Deep capillary plexus (DCP) slap in the OCTA demonstrates hypoperfusion area in DCP (green area), and segmentation defect/artifact (red area) mimics hypoperfusion area caused by intraretinal cystoid change (arrow head) which corresponded with (e) structural en-face image and (f) B-scan OCT image. (e) Structural en-face image of DCP slap demonstrates intraretinal cystoid change.

Perifoveal hypoperfusion area in OCTA was defined as the area within the inner border of terminal capillary ring and the area that >50% loss of vascular density [10] which were shown within 3×3 mm image centered at fovea. It was evaluated conjunction with corresponding structural en-face images and B-scan OCT images. Segmentation artifact either from intraretinal cystoid change, or retinoschisis, shown in structural en-face image and B-scan OCT images, was not included as a hypoperfusion area (Figures 1(a) and 1(f)). Hypoperfusion area was delineated using a drawing tool in IMAGEnet, and it was automatically calculated into square millimeter. Superficial capillary plexus (SCP) was defined as the vascular plexus shown in the slab between 3 *μ*m below the internal limiting membrane and 15 *μ*m below the inner plexiform layer. Deep capillary plexus (DCP) was the vascular plexus shown in the slab between 15 *μ*m and 70 *μ*m below the inner plexiform layer.

Hypoperfusion area in FA was measured 8 seconds after the dye was seen in the first central retinal artery bifurcation using the Heidelberg Retinal Angiogram-2 (HRA-2; Heidelberg Engineering GmBH, Dossenheim, Germany) [11]. It was delineated using a drawing tool in Heidelberg Eye Explore and was automatically calculated into square millimeter. Fluorescein activity of uveitis determined by capillary leakage was analyzed.

The grading of FRT and the area of hypoperfusion were made by 2 independent graders (SL and PC for FRT, OCTA, SL, and WK for FA), and any difference greater than 10% of the mean was corrected by open adjudication with 2 retina experts (TS and SV). Subgroup analysis of eyes with foveal thinning compare to the eyes with normal foveal thickness was performed. Thin FRT was defined by decrease in FRT more than 10% of normal FRT measured by SS-OCT in the prior study [12].

Descriptive data were presented with proportion and mean ± standard deviation. Wilcoxon Signed Ranks test was used to compare SCP and DCP hypoperfusion area. One-way ANOVA and independent *t*-test to compare FRT with VA and disease duration were used, while Kruskal Wallis test and Mann–Whitney *U* test for subcategorized correlation analysis.

## 3. Results

A total of 37 eyes in 21 consecutive Behcet's uveitic patients were included in the study. Five eyes were excluded: two eyes due to patients could not fix the eye during image acquisition and one each by dense cataract, macular scar, and blindness caused by retinal detachment, respectively. Eleven males and 10 females, with a ratio of 1.44 : 1, were participated. The mean age at presentation was 44.81 ± 12.93 years, while the mean age at the time of the first manifestation was 36.71 ± 12.57 years (range: 19–69 years). Mean duration of disease was 8.10 ± 7.54 (range: 1–25 years). Half of the eyes have visual acuity 20/50 or better, and 21 percent have visual acuity worse than 20/200. The mean intraocular pressure was 13.5 ± 3.40 mmHg. Cataract and vitreoretinal surgery were performed in 5 and 1 eyes, respectively. Demographic data and clinical characteristic were showed in Table 1. The major sequelae of ocular Behcet were cataract (64.86%), retinal atrophy (40.54%), and epimacular membrane (38.84%) (Table 2). Seven eyes (18.92%) had macular edema with intraretinal cystoid change, and none of macular edema patients had diffuse thickening without cystoid spaces. Twelve eyes (32.43%) of six patients had fluorescein activity. Fifty percent (6 eyes) of that do not have clinical activity of uveitis by slit-lamp examination.

The mean FRT was 118.1 ± 52.35 *μ*m. Mean hypoperfusion area defined by FA in 15 eyes was 0.65 ± 0.35 mm^2^. Using OCTA (*n* = 26 eyes), area of hypoperfusion in DCP (mean: 1.92 ± 3.76 mm^2^) was statistically significantly greater than that of hypoperfusion area in SCP (mean: 0.47 ± 0.17 mm^2^), *p* value < 0.001. Mean superficial capillary plexus to deep capillary plexus (SCP : DCP) ratio was 0.57 ± 0.26 mm^2^ (Table 3).

We found moderate correlation between FRT and visual acuity (*R*^2^ = 0.243, *p* = 0.003, 95% CI −60.47, −13.92). There is a moderate negative coefficient correlation between SCP : DCP ratio and poor visual acuity (*R*^2^ = 0.169, *p* = 0.041, 95% CI −0.644, −0.015) without correlation to disease duration. There is no difference neither using OCTA nor FA to define the perifoveal hypoperfusion area (Table 4).

In subgroup analysis of thin FRT (14 eyes), thin FRT had a statistically significant correlation with poor visual acuity (*p* = 0.009) but not disease duration (Table 5). Mean area of hypoperfusion in SCP and DCP was 2.67 ± 2.10 mm^2^ and 5.27 ± 3.78 mm^2^, respectively. There is no difference between the mean SCP : DCP ratio in thin FRT (mean: 0.47 ± 0.14 mm^2^) and normal FRT (6 eyes) without intraretinal cystoid change (mean: 0.30 ± 0.22 mm^2^), *p* value 0.57.

## 4. Discussion

Behcet is an inflammatory disease that affects small retinal vessel bilaterally. FA is the gold standard imaging technique to provide the information of nonperfusion area and identify foveal avascular zone (FAZ). The standard FAZ measurement is in the early phase of the FA study, which the investigator needs to choose one of the patient's eyes. The late phase of FA is not appropriate to measure either FAZ or nonperfusion areas due to leakage of the dye from the abnormal vessels that can be obscured and can lead to underestimate the area of nonperfusion. This is the limitation to measure FAZ and nonperfusion area in the bilateral disease like Behcet. Moreover, the standard FA measures over all retinal nonperfusion that cannot demonstrate SCP and DCP separately. However, subgroup analysis in our study showed that half of ocular Behcet eyes had fluorescein activity showing active uveitis. All of these showed no clinical activity of uveitis by slit-lamp examination. This emphasizes the essential role of conventional FA for disease activity evaluation in ocular Behcet.

The advantage of OCTA allows ophthalmologists to measure FAZ and nonperfusion area bilaterally without obscuration by leakage of fluorescein dye. The other major advantage is to evaluate inner retinal circulation (SCP and DCP), outer retinal circulation, and choriocapillaris, separately. In our study, we compare nonperfusion area, FAZ, measured by cSLO FA versus OCTA (Figures 1(a), 1(b), and 1(c)). We found that nonperfusion area defined by FA is larger than nonperfusion area in SCP but smaller than nonperfusion area in DCP measured by OCTA; mean areas of nonperfusion are 0.65, 0.47, and 1.92 mm^2^, respectively. Prior studies of OCTA in normal eyes shown that FAZ defined in DCP is larger than that in SCP with SCP : DCP ratio 0.53 [13]. Consistant with our series, hypoperfusion area in DCP is significantly greater than that in SCP. SCP : DCP ratio in ocular Behcet is also greater than prior report in the normal eyes. The segmentation artifact of the OCTA, such as artifacts by intraretinal cystoid change, was excluded for the nonperfusion area measured in our study (Figures 1(c) and 1(e)). These areas can be even perfusion or hypo/nonperfusion when the cystoids are resolved, and these excluded areas can cause underestimation of nonperfusion area of DCP loss measured in our study.

One of the well-known clinical manifestations of active ocular Behcet is cotton wool spots (CWS) caused by infarction of nerve fiber layer. Cotton wool spots tend to resolve without any clinical significant retinal change in 6–8 weeks. The other feature is extrafoveal faint intraretinal whitening lesion that looks deeper and less bright than CWS. Same as CWS, this lesion generally resolves without scar in 6–8 weeks. OCT scan through this deep lesion shows intraretinal hyperreflectivity in the inner nuclear layer and inner plexiform layer which is consistent with infarction of DCP [14]. The infarction of retinal vessel and increase area of DCP nonperfusion were reported in the other retinal vascular diseases such as diabetic retinopathy and retinal vein occlusion [15]. Our study in Behcet uveitis patients also showed that nonperfusion area in DCP is more severely involved than that in SCP, consistent with the study by Khairallah et al. [7]. Loss of DCP in OCTA tends to be found in the early stage of disease than that in SCP. DCP seems to be more vulnerable to ischemic process than SCP because DCP is the intraretinal watershed zone between inner and outer retinal circulation, and likely to be affected in the early stage of ischemic retinal vascular change. The other possibility is that the detection of retinal vasculature defined by OCTA is dependent on the detection of decorrelation signal from the moving structures, especially red blood cell in the blood column, either too high or too low flow that out of detection limit of OCTA can lead to misinterpret as nonperfusion. Too high flow is very unlikely to happen in the retinal vascular supply. The nonperfusion area defined by OCTA in our study is more likely caused by real nonperfusion or too low retinal capillary flow.

Previous studies in ocular Behcet showed that the vascular supply in the posterior pole tends to be spared until the late stage of the disease with generalized vascular occlusion form the prior vasculitis [16]. However, FA has the limitation to detect DCP and could underestimate macular ischemia due to the presence of cystoid macular edema or leakage of dye in the macular area. Our study showed that selective DCP loss from the OCTA even in the eye with normal FAZ demonstrated by FA and nonperfusion could happen in any stages of ocular Behcet especially in the DCP layer which can reflect the pathogenesis of central visual loss in Bechet's uveitis. Macular ischemia is one of the major causes of permanent visual loss in various retinal vascular diseases. The essentiality of immunosuppressive therapy for Behcet uveitis treatment even in the early stage of disease should be emphasized especially in the cases that compromise in DCP perfusion is detected in OCTA. OCTA could be the useful tool for early detection of poor macular perfusion and indicate prompt treatment. The previous theory which stated that retinal vascular involvement in the peripheral area manifests earlier than that in the posterior pole is challenged by our study. DCP loss might be the earliest sign, prior to SCP involvement, of macular ischemia and could explain the pathogenesis of Behcet diseases and progressive permanent visual loss in ocular Behcet, even if the FFA has no sign of leakage from the peripheral retinal vessels nor no cystoid macular edema in the conventional OCT.

The prior study by Mustafa et al. showed that mean FRT was thinner in eyes with Behcet's disease than in eyes of healthy controls [17]. This finding referred to permanent retina structural loss in the foveal area which is consistent with our study and found that thin FRT correlates with poor visual outcome. Furthermore, our study showed negative correlation between SCP : DCP nonperfusion ratio and poor visual acuity. This reflex degree of DCP loss is greater overtime through the stage and severity of ocular Behcet disease. However, in our subgroup, analysis of thin FRT showed no statistical significance in SCP : DCP nonperfusion ratio compared to normal FRT without intraretinal cystoid change group that might be due to small numbers of eyes in subgroup analysis. Limitations of our study are relative small number of participants, lack of control group, and the limitations according to the cross-sectional study design; future longitudinal study with long-term follow-up in various clinical stages of disease is required.

## 5. Conclusion

OCTA is an alternative noninvasive method to identify and monitor macular ischemia in ocular Behcet. Behcet's uveitis affects deep capillary plexus more than superficial capillary plexus. Decreased superficial to deep capillary plexus ratio and decreased foveal retinal thickness correlate well with poor visual acuity. Macular ischemia and selective loss of DCP can be the early signs and can explain vision loss in Behcet.

## Figures and Tables

**Table 1:** Demographic data and clinical characteristics.

Total number of patients (eyes)		21 (37)
Demographic data		
Gender (male)	*n* (%)	11 (52.40%)
Age (years)	Mean ± SD	44.81 ± 12.93
Age of onset (years)	Mean ± SD	36.71 ± 12.57
Duration (years)	Mean ± SD	8.10 ± 7.54
Ocular manifestation		
Best corrected visual acuity		
20/20	*n* (%)	6 (14.29%)
20/25–20/50	*n* (%)	16 (38.10%)
20/40–20/200	*n* (%)	9 (21.43%)
Worse than 20/200	*n* (%)	9 (21.43%)
Affected eyes (bilateral involvement)	*n* (%)	38 (90.5%)
Intraocular pressure (mmHg)	Mean ± SD	13.50 ± 3.40
Previous surgery (eyes)		
Cataract surgery	*n* (%)	5 (13.51%)
Vitreoretinal surgery	*n* (%)	1 (2.70%)
Glaucoma surgery	*n* (%)	0

**Table 2:** Ocular sequelae and complications (*n* = 37 eyes).

	*n* (%)
Cataract	24 (64.86%)
Thin foveal retinal thickness	15 (40.54%)
Epimacular membrane	14 (38.84%)
Posterior synechiae	13 (35.13%)
Macular edema	7 (18.92%)
Rising of intraocular pressure	6 (16.22%)
Glaucoma	5 (13.51%)
Others	5 (13.51%)

**Table 3:** Demonstrate perifoveal hypoperfusion area.

	Mean ± SD (mm^2^)	*p* value
Hypoperfusion area defined by FA (*n* = 15 eyes)	0.65 ± 0.35	
Hypoperfusion area defined by OCTA (*n* = 26 eyes)		
Superficial capillary plexus	0.47 ± 0.17	<0.001^∗^
Deep capillary plexus	1.92 ± 3.76	
Superficial to deep plexus ratio	0.57 ± 0.26	

^∗^Statistically significant using Wilcoxon signed ranks test.

**Table 4:** Correlations between OCTA hypoperfusion areas, visual acuity, disease duration, and hypoperfusion area defined by FA.

		Visual acuity	Disease duration	Hypoperfusion area in FA
Superficial capillary plexus	*R*	0.08	−0.24	0.13
	*p* value	0.70	0.42	0.68

Deep capillary plexus	*R*	0.12	−0.13	−0.16
	*p* value	0.58	0.67	0.62

Superficial to deep plexus ratio	*R*	−0.41	−0.06	0.12
	*p* value	0.04^∗^	0.84	0.71

^∗^Significant correlations (2 tails).

**Table 5:** Subgroup analysis of foveal retinal thickness with visual acuity and disease duration.

	*n*	Mean ± SD (mm^2^)
Visual acuity (*p* = 0.009^∗^)		
Normal (20/20–20/40)	6	186.50 ± 16.53
Mild visual impairment (20/50–20/70)	16	204.19 ± 40.33
Moderate visual impairment (20/100–20/200)	8	199.69 ± 64.74
Severe visual impairment (>20/200)	5	118.50 ± 53.17
Duration (*p* = 0.99^†^)		
≤5 years	12	204.63 ± 49.81
>5 years	6	205.17 ± 45.17

^∗^Statistically significant (one-way ANOVA).

^†^Independence *t*-test.
